# Supplementary material for: Understanding school-going adolescent’s preferences for accessing HIV and contraceptive care: findings from a discrete choice experiment among learners in Gauteng, South Africa
Source: BMC Health Serv Res. 2023 Dec 8;23:1378. doi: 10.1186/s12913-023-10414-w (PMC10704722; doi:10.1186/s12913-023-10414-w)
Supplement: Supplementary file 1 — Additional file 1: Figure a1. Detailed description of DCE attributes and respective levels, Figure a2. Flow chart of participants for the DCE by school [file 12913_2023_10414_MOESM1_ESM.docx]

**Figure a1. Detailed description of DCE attributes and respective levels**

| **Attribute** | **Level 1** | **Level 2** | **Level 3** | **Level 4** |
| --- | --- | --- | --- | --- |
| **Location** | **Clinic** | **School-based** | **Community/door to door** | **Private (doctor or pharmacy)** |
|  | This is the traditional primary health care facility, government funded, offering basic primary health services | Health-services offered at secondary school | Health services that come to your community. This includes mobile clinics, home visits by community health workers, or services available in shops, taverns and salons. | Care from a private doctor or pharmacist |
| **Operating times** | **Weekday morning (until 12pm)** | **Weekday afternoon (until 4pm)** | **Weekday evening (4-8pm)** | **Weekends** |
|  | Health services available Monday to Friday in the morning  from 7.30am to 12pm | Health services available Monday to Friday in the afternoon 12 to 4pm | Health services available outside traditional clinic hours. Available weekday evenings 4 to 8pm | Health services made available during the day on Saturday and/or Sunday |
| **Health-care provider (HCP) characteristics** | **Young HCP from within community** | **Young HCP from outside community** | **Older HCP (>40yrs) from within community** | **Older HCP (>40yrs) from outside community** |
|  | This health care provider is a young trained health care provider from within your community | This health care provider is a young trained health care provider who comes from outside your community | This health care provider is an older person (e.g. greater than 40 years old) from within your community | This health care provider is an older person (e.g. greater than 40 years old) from outside your community |
| **Staff attitude** | **Friendly, open and welcoming** | **Unfriendly and judgmental** |  |  |
|  | This health care provider is friendly, open and welcoming, who listens to your problem and is not judgmental | This health care provider is not very welcoming, they can be unfriendly and judgmental about what you are telling them |  |  |
| **Confidentiality of services** | **Not very confidential** | **Highly confidential** |  |  |
|  | Health-services are often provided where others can see you and hear what is being discussed with you | Health services are provided in a quiet, private and confidential space where no one else can see and hear |  |  |
| **Incentives** | **None** | **Youth only waiting area and services** | **Free Wifi** | **Food cheap and easily available** |
|  | There are no additional incentives to using these services other than the health services provided | These health services have youth friendly waiting areas where only young people can wait and/or youth friendly services | There is free wifi that can be used by anyone waiting for these services | There is food that is cheap and easily available for purchase where these services are delivered |
| **Type of services offered** | **Condoms only** | **Contraceptive and family planning services only** | **HIV testing and counselling services only** | **All health services (including HIV and contraceptives)** |
|  | Only condoms are available where these services are provided | You can get all types of contraceptives (e.g. male and female condoms, the loop, injectable contraceptives, the pill) where these services are provided | These services only provide HIV counselling and testing services | All health services are available including all HIV services as well as contraceptive and family planning services |
| **Cost including travel** | **Free** | **ZAR10-50** | **ZAR51-100** | **More than ZAR100** |
|  | It does not cost you anything to access these services | To use these services will cost you between ZAR10 and R50 including your travel costs | To use these services will cost you between ZAR51 and R100 including your travel costs | To use these services will cost you more than R100 including your travel costs |

**Figure a2. Flow chart of participants for the DCE by school**

Consent forms given out (n=2245)

School 1 (n=112, 14%)

School 2 (n=142, 18%)

School 3 (n=81, 10%)

School 4 (n=112, 14%)

School 5 (n=70, 9%

School 6 (n=85, 10%)

School 7 (n=40, 5%)

School 8 (n=67, 8%)

School 9 (n=26, 3%)

School 10 (n=70, 9%)

Consent forms returned (n=1068, 47%)

Attended DCE (n=805, 75%)
