# Supplementary material for: Understanding school-going adolescent’s preferences for accessing HIV and contraceptive care: findings from a discrete choice experiment among learners in Gauteng, South Africa
Source: BMC Health Serv Res. 2023 Dec 8;23:1378. doi: 10.1186/s12913-023-10414-w (PMC10704722; doi:10.1186/s12913-023-10414-w)
Supplement: Supplementary file 2 — Additional file 2. [file 12913_2023_10414_MOESM2_ESM.docx]

**The CHoiCE Study - The preferences of high school students in South Africa for HIV and contraceptive services: The design and implementation of a discrete choice experiment**

**Introduction**

Thank you for taking the time to do this survey with us. As explained on your information sheet, the reason we are doing this survey is to try and understand what young peoples’ preferences are when deciding whether or not to use health care services and when accessing contraceptive and HIV health services. **Please remember that this survey is anonymous and that it will not be possible to link any of your answers to you as an individual as your name is not recorded on any of the answer sheets.**

The survey will be divided into three sections.

1. The first section will ask some personal questions. There will be some questions about you, your partners and sex life, HIV testing history and use of contraception. We know that this information is personal and private and it is possible that some of these questions may make you feel uncomfortable. There are no right or wrong answers and you do not have to answer any questions if you do not wish so, but remember we have no way to link your answers to you and we would encourage you to answer all questions completely and honestly.
2. In the second section we ask you to identify which aspects of HIV and contraceptive services are most important to you when deciding whether or not to access those services, and which are least important.
3. The final section of the questionnaire is where options between two types of service will be given. We will ask you nine questions. For each question there is a table with a choice of two service options. You will need to identify which option you would prefer. The second question on each page then asks whether you would use the option that you have chosen if it was available. Again please remember your answers are anonymous and there are no right or wrong answers.

**Instructions**

A study team member has given you an answer sheet which you should use to record all your answers. This answer sheet will be pink, blue, green or yellow. The answer sheet has a Study ID which helps us make sure that answers from one person stay together. We cannot identify you from this number and please **DO NOT** write your name anywhere on this answer sheet.

To answer a question please colour in the appropriate circle on the answer sheet. For example if your answer is option A please colour the circle labelled ‘A’. PLEASE DO NOT WRITE ANY ANSWERS ON THIS SHEET.

You have been divided into four separate blocks (Pink, blue, green, yellow). The questions for the last section - Section C – are slightly different for each group. It is important that the colour of your answer sheet matches the colour of this questionnaire book i.e. if you have a pink answer sheet you should have a pink questionnaire book. Please let a member of the study team know if your answer sheet and questionnaire are different colours. PLEASE ONLY COMPLETE YOUR ANSWERS ON THE ANSWER SHEET, PLEASE DO NOT WRITE ANYTHING ON THE QUESTIONNAIRE BOOK. To help you complete the final section, we will describe the different services and levels of service options that you will see in these choice options and the pictures that are used after this introduction.

Once you have completed the survey please hand your answer sheet and question book back to one of the study team. You are then free to leave.

If you have any questions or want to stop at any time just let a member of the study team know.

**These are the pictures that will be used in the final section of the questionnaire. Each picture represents a level of the attribute (characteristic). Each choice (option) will contain a level from each attribute.**

**Section A: Individual characteristics**

Colour in the appropriate circle on the answer sheet. **PLEASE DO NOT WRITE ANY ANSWERS IN THIS BOOK.**

|  |  |  |
| --- | --- | --- |
| 1. How old are you? | A | 15 years |
|  | B | 16 years |
|  | C | 17 years |
|  | D | 18 years |
|  | E | 19 years and older |
|  |  |  |
| 2. What grade are you in? | A | Grade 9 |
|  | B | Grade 10 |
|  | C | Grade 11 |
|  | D | Grade 12 |
|  |  |  |
| 3. What is your gender? | A | Male |
|  | B | Female |
|  | C | Other |
|  |  |  |
| 4. What is your most common home language? | A | English |
|  | B | Xhosa |
|  | C | Tswana |
|  | D | Zulu |
|  | E | Sotho |
|  | F | Ndebele |
|  | G | Other |
|  |  |  |
| 5. Do you receive any pocket money or earn any money each month? | A | No |
|  | B | ZAR10-50 per month |
|  | C | ZAR51-100 per month |
|  | D | More than ZAR100 per month |

| 6. When you go to the clinic who do you prefer to see? | A | Nurse |
| --- | --- | --- |
|  | B | Doctor |
|  | C | Lay counsellor |

| 7. What is the best way for you to get information about health services? | A | Social media (e.g. WhatsApp, Facebook, Snapchat, Twitter) |
| --- | --- | --- |
|  | B | SMS messages |
|  | C | Printed materials |
|  | D | TV or radio |
|  | E | Support groups |
|  | F | Health care provider |
|  | G | Teacher |
|  |  |  |
| 8. Have you ever used any of the following services? | A | Contraceptive services |
|  | B | Family planning services |
|  | C | Antenatal care |
|  | D | Birth/delivery services |
|  | E | Sexually transmitted infections testing/treatment |
|  | F | HIV testing |
|  | G | HIV treatment |
|  |  |  |
| 9. If it was easy to access condoms at different locations would you access them? | A | No |
|  | B | Yes |
|  |  |  |
| 10. If contraceptive services were easily accessible to you would you access them? | A | No |
|  | B | Yes |
|  |  |  |
| 11. If HIV testing and treatment services were easily accessible to you would you access them? | A | No |
|  | B | Yes |

| 12. Have you ever had sex? | A | No |
| --- | --- | --- |
|  | B | Yes |
|  |  |  |
| 13. How many sexual partners have you ever had? | A | None |
|  | B | One (1) |
|  | C | 2 to 4 |
|  | D | 5 or more |
| 14. How many sexual partners do you currently have? | A | 0 - I don' t have a partner |
|  | B | One (1) |
|  | C | 2 to 4 |
|  | D | 5 or more |
|  |  |  |
| 15. Have you accessed or tried to access HIV or contraceptive services in the last 12 months? | A | No |
|  | B | Contraceptive services only |
|  | C | HIV services only |
|  | D | Both HIV and contraceptive services |
|  |  |  |
| 16. Have you ever used condoms? | A | No |
|  | B | Yes |
|  |  |  |
| 17. Did you or your partner use a condom last time you had sex? | A | Yes |
|  | B | No did not use condom |
|  | C | No sexual partner |
|  |  |  |
| 18. Have you or your partner ever used other contraceptives? | A | No did not use other contraceptive |
|  | B | Depo/injectable |
|  | C | Implant |
|  | D | Oral contraceptive pill |
|  | E | Coil or loop |
|  | F | Traditional contraceptive |
|  | G | Other |
|  | H | No sexual partner |
|  |  |  |
| 19. Have you ever tested for HIV? | A | No |
|  | B | Yes |
|  |  |  |
| 20. Have you tested for HIV in the last 12 months? | A | Never tested |
|  | B | Have not tested in last 12 months |
|  | C | Yes |
|  |  |  |
| 21. Do you think that you (or your partner) is at low or high risk of getting pregnant? | A | No risk |
|  | B | Low risk |
|  | C | High risk |

|  |  |  |
| --- | --- | --- |
| 22. Do you think that you are at low or high risk for HIV? | A | No risk |
|  | B | Low risk |
|  | C | High risk |
|  |  |  |
| 23. Do you think that you are at low or high risk for getting a sexually transmitted infection e.g. gonorrhoea, chlamydia? | A | No risk |
|  | B | Low risk |
|  | C | High risk |

**Section B: Ranking of attributes related to health care services**

The following are characteristics that other youth have identified as important when deciding whether or not they will use health care services.

| 24. Which of these characteristics is **most** important to you when deciding where to access health care services. | A | The location of the service |
| --- | --- | --- |
|  | B | The times that the services are available |
|  | C | The age of the health care provider and where they are from |
|  | D | Whether the health care provider is friendly or not |
|  | E | Confidentiality and privacy |
|  | F | Incentives |
|  | G | The types of service that is available at the location |
|  | H | The cost of getting to the service |

| 25. Which of these characteristics is **least** important to you when deciding where to access health care services. | A | The location of the service |
| --- | --- | --- |
|  | B | The times that the services are available |
|  | C | The age of the health care provider and where they are from |
|  | D | Whether the health care provider is friendly or not |
|  | E | Confidentiality and privacy |
|  | F | Incentives |
|  | G | The types of service that is available at the location |
|  | H | The cost of getting to the service |

| 26. Which of these locations would you **prefer** if you needed HIV or contraceptive services? | A | Clinic |
| --- | --- | --- |
|  | B | School |
|  | C | Community or door-to-door services |
|  | D | A private doctor or pharmacy |

| 27. Which of these operating times would be **best** for you if you needed HIV or contraceptive services? | A | Weekday mornings (7.30am – 12pm) |
| --- | --- | --- |
|  | B | Weekday afternoons (12-4pm) |
|  | C | Weekday evenings (4-8pm) |
|  | D | Weekends |

| 28. Which of these health care providers would you **prefer** if you needed HIV or contraceptive services? | A | Young health care provider from within community |
| --- | --- | --- |
|  | B | Young health care provider from outside community |
|  | C | Older health care provider (>40yrs) from within community |
|  | D | Older health care provider (>40yrs) from outside community |

| 29. Which incentive would most encourage you to access HIV or contraceptive services? | A | Youth only waiting area and services |
| --- | --- | --- |
|  | B | Free Wi-Fi |
|  | C | Food that is cheap and easily available |

| 30. Which of these health services are most important for you to be able to access easily? | A | Condoms only |
| --- | --- | --- |
|  | B | Contraceptive and family planning services |
|  | C | HIV testing and counselling services |
|  | D | All health services (including HIV and contraceptives) |

| 31. If you had to pay for any of these services, what amount would you consider reasonable to pay to travel to and access these services? | A | ZAR1-50 |
| --- | --- | --- |
|  | B | ZAR51-100 |
|  | C | More than ZAR100 |
